# Clinique Chirurgicale, exercé particulierement dans les Camps, et les Hôpitaux Militaires depuis 1792, jusqu' en 1829

**Published:** 1831

**Authors:** 


### Medico-Chirurgical Review,

### No. XXIX.

APRIL 1, TO JULY 1, 1831.

### I.

Clinique Chirurgicale, exercé particulierement dans les Camps, et les Hôpitaux Militaires depuis 1792, jusqu' en 1829. Par *Le Baron Larrey*, Membre de l'Institut de France. 3 Volumes 8vo. with a Volume of Plates. Bailliere, 1830.

In an age of books—when every cradle sends forth an author—when those who write are scarcely surpassed in number by those who read—and when, notwithstanding all this *cacoethes scribendi*, we are doomed to wade over twenty volumes, before we meet with one which repays the purchase-money, or is worth enrolling in our private catalogue—it is refreshing to peruse such a performance as the *Clinique Chirurgicale.* Unlike the provincial Essays of our lane-and-hedge observers, in which a few fancies are concocted by a strong imagination into the form of facts, and artfully spread over an extended surface, that however they may lack in matter they may not be confined in mould—it teems in rare and weighty knowledge, and every position, fact and statement are communicated through the medium of cases, which are too circumstantially recorded to be suspected of inaccuracy.

It fell to the happy lot of Larrey, to become the intimate friend and field-companion of the most enterprising warrior, that ever ransacked empires and shattered thrones—to follow him in all his flights of victory—to accompany him in all his journeys of ambition—to fight with him in all his battles—to share with him in all his fortunes—to see every variety of disease in almost every country, and to treat every form of accident under the influence of almost every climate. Witness of twenty-six campaigns in the four quarters of the globe—whether stationed amid the burning sands of Egypt, or the frozen wilds of Russia—wherever the eagles of Napoleon, or his armies flew—wherever the cannon and the sword were sent to sweep thousands to their graves—there was the active and philanthropic Larrey, stemming the tide of human blood—husbanding the waste of human life—pouring oil and balm into the wounds of his countrymen—diminishing the havoc of outrageous war—and gathering a fund of observations for the advancement of medical science, which perhaps no former period of the world offered such splendid opportunities of collecting, and for the accumulation of which perhaps no preceding surgeon was more unexceptionably qualified.

It may easily be imagined, therefore, that from a volume, of five hundred pages, emanating from such a source, no single article, however long or laboured, could extract every important fact, or review every interesting sentiment. Some observations must be overlooked, and some statements must remain uncriticised; but as the present is the first of these volumes, to each of which equal attention is indispensable, the most striking novelties only have been culled, and less important matter has been referred to.

*Diseases of the head* occupy the principal portion of the present volume, and it is mainly to the consideration of these affections that our attention shall be confined; but as some observations, of considerable interest, are contained in the Baron's sketch of the nature and treatment of wounds in general, as well as in his views of tetanus, it might not be well wholly to overlook them. While speaking of wounds produced by the bite of a rabid animal, he takes occasion to state his opinion as to the nature of *hydrophobia.*

"It is difficult," he says, "to explain how the virus of a rabid animal can remain latent in the system for a long time, can develop itself afterwards, and can end by producing the most terrible effects. It would, nevertheless, appear that this subtle and unknown poison has a particular partiality for the nerves, and concentrates itself by preference in the nervous system; in which it can remain inactive for a lapse of time more or less considerable, on an average, as we have said, of thirty or forty days. Its effects, when it is developed, are purely nervous, and it is this fact which seems to justify this assertion."

It cannot be denied but that the most evident indications of inflammatory action attend the symptoms, and distinguish the pathology of hydrophobia —that we have often inflammation of the œsophagus, pharynx and larynx, and occasionally of the brain and spinal cord; yet it is generally admitted, that these appearances are more the consequences than the cause of the disorder, and that although frequently present with, they are by no means essential to, the existence of hydrophobic action. Rossi of Turin has performed experiments, which, were they sufficiently confirmed, must be regarded as strongly corroborative of Larrey's theory. He dissected out a portion of nerve from the body of a rabid animal, during the violence of its paroxysm, and making an incision into the flesh of a healthy animal, inserted this extracted piece of diseased nerve into the incision. After some time, the animal, thus inoculated, became equally mad with the one from which the poisoned nerve had been received, and died affected with the same symptoms. The following case will be read with interest, and strongly favors the author's doctrine—that hydrophobia is purely a nervous disorder. A soldier, æt. 20, had his thigh bit by a mad dog, when a youth of between fourteen or fifteen years of age. From this period, until he came under the care of Larrey, Courmontague did not cease to experience a kind of nervous affection, which was characterized by spasms and a slight aberration of the intellectual faculties. He was irritable, very loquacious, and was frequently agitated by automatic movements. He was emaciated, his eyes were haggard, he was often afflicted with vertigo and dimness of sight, and he felt an invincible repugnance against the sight of limpid fluids, even in circumstances in which his companions, oppressed by heat, were compelled to drink in his presence. He drank bitter ptisans and other coloured liquids with more, or less avidity. These were his principal symptoms when he came under the management of Larrey—not on their account—but for a sprain of the right foot, which he had received during violent exertion. To this affection were soon superadded symptoms of nostalgia, and Courmontague expressed a strong desire to be dismissed the service. Obstinately bent upon this purpose, far from allowing his cure to proceed, he secretly employed means to swell the leg and to aggravate the disease; so that in a short time gangrene appeared in the inside of the foot, and spread so hastily that the whole limb became sphacelated, and was amputated. After some attacks of traumatic irritation, caused by deviations from the prescribed regimen, two-thirds of the wound had cicatrized, when, on the 30th day after the operation, the patient suddenly refused all kinds of transparent liquids, and became attacked with signs of cerebral inflammation. He was convulsed, he locked his jaws, he ground his teeth, and fell into a complete state of tetanic contraction. All the excretions were suppressed, the spasm and rigidity increased, and during the night of the 33d he expired. On inspection were found hypertrophy of the cranium, principally in the occipital region, considerable engorgement of the vessels both of the membranes and brain, of the superior longitudinal sinus and plexus choroides; slight granulations on the surface of the hemisphere; about an ounce of yellow serum in the lateral ventricles; firmness and density of the whole brain, of the spinal cord, and especially of the tuber annulare, in the substance of which a red tinge was evidently manifest; and the neurilema of the spinal nerves was stained of the same colour. The mucous membranes were all healthy; except a few old adhesions, nothing morbid could be detected in the lungs; and all the viscera of the abdomen presented a natural appearance. The pericardium was firmly attached to the heart in its whole extent, but evidently by adhesions of a former date; its cavities were very contracted, and the great vessels, which issued from it, were not more than two-thirds of their ordinary size.

It is tolerably evident, from the history of this case, both before and after death, that the first and last symptoms, by which it was distinguished, were referrible to the wound occasioned by the bite of the mad dog; and that the poison, which had been thus inserted in the system, remained comparatively latent for the period of six years, giving rise to some anomalous nervous symptoms only, which scarcely if ever amounted to disease, until meeting with an exciting cause, which awoke this dormant principle into full activity, the patient was no longer respited from its fatal consequences. There are very many facts, however, in existence relative to hydrophobia, which in our minds distinguish it clearly from ordinary tetanus; and unless there was much stronger reason than there is for identifying these disorders, no one should make the attempt, as it is only calculated to lead the unthinking into a state of carelessness, with reference to what is now generally considered the almost exclusive cause of this formidable malady.

There is nothing in the Baron's observations on the treatment of *punctured*, *lacerated*, or *poisoned wounds*, which may detain us. In *traumatic erysipelas* he is a strong advocate for the application of the actual cautery. According to him it scarcely produces any pain, it diminishes the heat, redness and tension of the affected parts, it is never followed by gangrene or suppuration, but ends in small dry eschars, which leave no sensible cicatrix, and it rapidly improves every symptom. How it effects all this he labours to explain, but his *modus operandi* is much more tedious than satisfactory. In spontaneous erysipelas he has found the same remedy equally beneficial; and whether the sovereign efficacy of the iron precludes the necessity of every other measure we know not, but certain it is, that the worthy author has not condescended even to hint at our humble efforts to establish a *coups de main* treatment of our own. Thirteen-inch incisions are apparently as little thought of across the Channel as they are at present here, and although we should be sorry to see the scalpel or needle prohibited in every case of erysipelas, convinced as we are of their utility in many, yet it may be as well both for their patients and themselves, that the primitive proposal of our English surgeon stands as little chance of adoption by the French, as that of the French surgeon probably does by the English. The fallen fate of this celebrated proposal has been obviously accelerated by the injudicious manner in which it was advanced; and as truth has frequently been scouted as error, because intemperately advocated, so real improvement may totally miscarry, by being brought forward with unnecessary parade and defended with dogmatic obstinacy.

The contagious character of *hospital gangrene* Larrey feels no scruple in maintaining; and if extensive observation can enhance experience, his testimony to the fact cannot with ease be set aside.

"When wounds, that have been far advanced towards healing, have been inoculated with matter from a sore affected with this disease, they have put on the same appearances, and have been converted into putrid ulcers. The linen, with which such tainted sores are surrounded, and the instruments employed in dressing them, are faithful conductors of the poison. The inoculation has been performed under my eyes, I have watched its progress through every stage, and, however it may oppose the sentiments of others, I entertain no doubt of the contagious nature of this affection."

It is generally, he admits, idiopathic, but, in opposition to the Professor of Montpellier, he considers that occasionally it is symptomatic of a deranged condition of the gastric organs. In such cases an emetic, sulphate of quinine, camphor, opium, and light mineral acids, will arrest its progress, dissipate the local irritation, and restore the wound to its originally healthy state. In idiopathic cases, local treatment is more necessary. The *eau de Labarraque* is one of his most favourite applications, and after the sore has been thoroughly deterged by this fluid, the actual cautery, in a state of incandescence, is liberally applied. This formidable weapon finds no equal in the choice of Larrey. Wherever the secretions are deranged, or the granulations are unhealthy; if the sore be indolent and too slowly heals, or be too active and becomes luxurious, *le cautère actuel* is the safest, speediest, and most sovereign remedy. It diminishes pain, dissipates inflammation, cleanses the wound, rectifies the granulations, recalls the secretions, and confers upon the patient "an inexpressible calm." Deducting our *granum salis* from this highly wrought estimate, and making all due allowance for paternal partialities for a favourite remedy, the actual cautery stands recommended to our regard by more than an average weight of unexceptionable evidence, and although a few prejudices have as yet to be overcome, we look forward to a period which, we imagine, is not far distant, when this Gallic weapon, barbarous as it may at first appear, shall be wielded with perhaps more discrimination, but not less faith by our English surgeons. If our acids and our alkalies, our nitrates and our sulphates, could achieve all that the actual cautery effects, the French had, long ere this, arrived at a discovery, which would have dispensed with a remedy so obnoxious to human feelings; and when the instantaneousness of its action is balanced with its intensity, there is reason to doubt whether the *actual* is more severe than the *potential* cautery.

In common gangrene this application is not recommended. While it alleviates and arrests the contagious form, it extends and aggravates the latter. Contagious gangrene is more a local, common gangrene more a general disease, and therefore less likely to be cured by topical remedies. While the French were in Egypt they were sadly persecuted by this affection. Even the slightest wounds, inflicted by the purest instruments, were attacked by gangrene; and those, who were seized by yellow fever while labouring under any solution of continuity, scarcely if ever escaped.

Larrey's description of *tetanus* is so excellent, that nothing but want of space prevents us from extracting it. He has found it especially to invade the young during great vicissitudes of temperature, and such as were of a dry, irritable temperament. Fear of limpid fluids he states as a frequent symptom, and the absence of all intellectual disturbance, while the disease seems to revel in the nervous system, he is inclined to explain by believing that the nerves are not prolongations, but perfectly independent of the brain. In emprosthotonos he has observed that the nerves usually affected lie on the anterior part of the body; in opisthotonos on the posterior part; while in complete and universal tetanus both sets are equally implicated. After the battle of Waterloo, he had many opportunities of inspecting the remains of those, who perished from tetanus in the hospitals of Louvain; and "invariably there were discoverable evident traces of inflammation on the spinal cord, with more or less effusion of a reddish colour within the sheath." Hence does he advise moxa, cupping-glasses, anodynes, and such applications to the spine as appear calculated to prevent, or remove the cause of irritation. Internal remedies of every description, if they are not always useless, are soon rendered so by the patient's falling into a state of strangulation shortly after he is attacked. While he can swallow, opium, camphor, musk, castor and alkalies may be given, and they are least obnoxiously given in the form of emulsion. The case of a Mameluke, belonging to Maurad Bey, is given, who was cured by internal medicines and warm-baths. Eight grains of camphor, the same quantity of musk, and twenty grains of opium were dissolved in a glassful of common emulsion, and the half was given for a dose. A few minutes after this draught was taken, the pains were diminished, the jaws relaxed, and sleep returned towards night. Next morning the patient felt much better, suppuration of the wound was re-established, the remainder of his medicine was taken as a second dose, all went on favourably, and he proceeded along his journey in return to his prince. Friction with oil, which some have so liberally extolled, he has not found to do any good; cataplasms made with the leaves of tobacco have been fruitless, and mercurial unguents produced only evident evil. "The employment of this last remedy," he observes, "even against syphilis, requires in Egypt the greatest care; for this mineral, administered in that climate as it is in Europe, has occasioned the worst consequences, among which we may enumerate madness and diseases of the liver." When tetanus appears after the wound has healed, cutting freely down upon the cicatrix is strongly recommended; but if after a ligature has been applied, it is probable that some nerve is included, and the ligature should be removed. In all compound fractures and dangerous wounds of the extremities, complicated with tetanic symptoms, the Baron's only sheet-anchor is the amputation-knife. From the success, which he has experienced in removing tetanus by amputating the wounded part, he has been encouraged to make the following proposal. "If, in tetanus, induced by such a wound of the extremities, as would not on its own account demand amputation, it be not better to remove the limb immediately tetanic symptoms appear, than to expect from the resources of nature and most uncertain remedies a very doubtful cure?" We cannot enter into his defence of this proposal, which merits serious consideration; but he concludes it, by saying that, "although I regret not having a greater number of recoveries to attest the value of this plan, I yet have sufficient to conclude that amputation, *faite à propos*, appears to me the most certain means of arresting and removing the effects of tetanus, which depends on a wound situated upon the extremities."

The Baron's favourite caustic is no mean remedy in tetanus, as well as gangrene, and the recovery of the patient, with whose case we shall conclude these observations upon this disease, which the author considers *one of the most remarkable in military surgery*, cannot certainly be ascribed to any inferior agent. A soldier was struck by a ball, which carried away a part of the spine belonging to the right scapulum, a portion of the trapezium, the sub and supra-spinati muscles. The shattered tendons were cut away, the lacerated pieces of muscles were removed, some splinters of bone were extracted, and the wound was carefully dressed. All went on well for some time, and the circumference of the wound began to cicatrize, when symptoms of tetanus suddenly appeared, and in a few hours after their appearance complete opisthotonos was established. Diaphoretic fluids and large doses of opiates were administered; oily, camphorated, and narcotic lotions were applied over the body. The purulent secretion being suspended the cicatrix made rapid progress, and in forty-eight hours it covered the half of the wound. At this period the patient felt in the cicatrized part a painful sensation, as though the edges of the wound had been enclosed between a pair of pincers; and the slightest pressure upon the delicate cicatrix, more especially of metals, as iron or steel, obliged him to emit piercing cries. Every tetanic symptom gradually increased, the superior extremities became rigid and were turned backwards, the cervical vertebræ were completely pulled down, and the power of deglutition was lost. In vain were two incisive teeth extracted, in order that medicines might be introduced into his stomach; not a drop got into the œsophagus, and the mere sight of pure water induced the most frightful convulsions. He foamed freely at the mouth, every symptom heightened, and this wretched man, whose bodily agony was ten-fold enhanced by a complete consciousness of his situation, had nothing to all appearance before him, but the prospect of a sad and sudden death.

When the disease had arrived at this crisis, Larrey resolved on trying the actual cautery.

"With this view," says he "I made red, even to incandescence, four large cauterizing irons, and I applied them in succession over the whole extent of the wound, so as to bear more strongly over those points of the cicatrix, where I

suspected the principal branches of the accessary nerve of Willis (spinal nerve) to be compressed and swollen. This application was extremely painful, nevertheless I had firmness enough to continue the operation, until all the surface of the wound was deeply and completely seared. Scarcely was this effected when a general stop was put to the progress of the disease. The patient voluntarily sat up and asked for drink, and the jaws separated spontaneously. Sixty drops of laudanum, and a few drops of Hoffman's mineral liquor were given him in a glassful of almond emulsion, in which a little nitre had been dissolved; the narcotic and camphorated liniments were repeated and the body was enveloped in warm flannel. The skin perspired profusely, a perfect calm came on which was succeeded by a deep sleep, and the next morning I found my patient free from every tetanic symptom."

The future dressings of the wound were simple, the eschar separated in nine or ten days, cicatrization recommenced speedily and without pain, and, beyond a little stiffness in the right arm and shoulder, Demoré was completely restored to health.

Larrey's views of *wounds of the scalp* and his treatment of injuries of the scull exhibit no peculiarity. Sutures, in the majority of cases, he strongly reprobates, the lightest dressings he particularly recommends, and, unless some urgency occur which requires these dressings to be removed, he advises them to be left undisturbed during the first seven days after the infliction of the injury. The latter part of this advice is extremely suspicious, and should not, we apprehend, be implicitly adhered to; for the natural secretions of the wounded part, accumulating and putrefying for such a period, could not fail to irritate and inflame the sore. What, therefore, might be gained by uninterrupted quiet, would probably be lost by want of cleanliness.

When the solution of continuity has passed through the integuments of the head, and proceeded to the brain, the symptoms vary with the part disturbed. If the anterior or superior extremities of the hemispheres have been injured, the Baron has generally observed that some sense is destroyed, or some intellectual function is deranged. Whereas, if the cerebral injury be limited to the base of the encephalon, no mental aberration can be perceived. In such instances different paralytic symptoms appear among the muscles, and the organ palsied invariably belongs to the side of the body opposite to that on which the injury was received. "These two principles," says the author, "suffer no exception, notwithstanding what has been asserted to the contrary;" and as several very interesting cases are detailed in their defence, it cannot be said, by such as consider them untrue, that they are likewise unsupported.

A soldier received the stroke of a sabre on his forehead over the coronal suture, which occasioned slight compression of the anterior lobes of the brain. In addition to the inflammation, which followed this injury, and which was subdued by the usual remedies, the only symptom which marked the progress of the cure was a very manifest incoherence in the ideas of the patient. His answers, although sometimes precise, had more frequently no reference to the questions proposed; but by proper care he was dismissed from the hospital, after six weeks, in all respects well.

Another soldier was similarly wounded on nearly the same place, and was brought under Larrey's care. On making out the history of his complaint, it was discovered that he had, on different previous occasions, received several injuries on the anterior part of the head, and that from that period to the present time his intellect betrayed some traces of derangement.

A grenadier was shot by a ball, which buried itself in the mastoid process of the left side of the head. Hemiplegy of the right side came on and he died in a state of lethargic torpor.

Nicholas Baumgartner, in an engagement with one of his companions, while under the influence of wine, received a blow on the left eye, and a part of the rod, with which it had been inflicted, was left behind in the orbit. Palsy of the left side came on, and although the wound and the hemiplegia occupied the same side, the Baron confided with so much confidence on the accuracy of his principle, that, in opposition to appearances, he referred to the right hemisphere as the principal seat of injury. No mental aberration was discoverable. The patient, on the contrary, was quite sensible of his situation, and replied to every question always rationally, although sometimes by signs, as he occasionally experienced some difficulty in articulating. Two days after the receipt of the injury he died, and, as had been anticipated, a small piece of wood, some lines in length, was found embedded in the middle lobe of the right hemisphere, near the fissure of Sylvius.

The following is an extremely instructive case. During the Russian campaign, a young grenadier received a wound from a lance on the posterior part of the head, towards the centre of the lambdoidal suture. The weapon was so well tempered that it penetrated far into the left posterior lobe of the brain, without producing any serious injury to the bone. The wounded man was left for dead upon the field, but being afterwards carried to a neighbouring village, his wound was dressed and ultimately healed, leaving, however, the patient deprived of most of his senses, and seriously impairing the functions of many of his internal viscera. The voice, after having been at first hoarse and obscure, was gradually lost, hearing, taste and smell were weakened, and the muscles of the larynx being partly paralyzed, this organ sunk down for half an inch below its natural position, in consequence of which the glottis was constricted, and the epiglottis curved down upon the rima by the irregular action of the muscles by which this cartilage is moved. In order to respire, the patient was compelled to clash his jaws firmly together, so as to raise the larynx by a simultaneous contraction of the attollent muscles of the pharynx and lower jaw, just as frogs do when swallowing air to fill their lungs. The diaphragm, participating in this paralysis, could no longer act upon the lungs: the pharynx, œsophagus and stomach had equally suffered. The abdomen did not alternate in its movements with the expansion and collapse of the chest, and the slightest exercise covered the body with perspiration, and rendered the face livid. His pulse was almost imperceptible, the motion of his heart could with difficulty be recognized, and his extremities were habitually cold. The intellectual functions, on the other hand, were quite untouched, and as he was unable to reply by speech to the questions he was asked, he wrote his answers with great precision and accuracy. "All these facts," argues the Baron, "are in my opinion strongly corroborative of Dr. Gall's view, that the reasoning faculties reside in the periphery of the superior and anterior portions of the brain." This fact we believe is now very generally admitted, but the author proceeds farther, and gives some countenance to the ridiculous detail, into which the advocates of phrenology have imprudently entered.

"Differences," he observes, "more specific than any of those, which have yet been mentioned, are submitted to our observation in cerebral symptoms, occasioned by mechanical or morbid lesions, which certain portions of the brain have sustained. When these injuries are confined to the cerebellum, the organs and functions of generation are especially affected, and when the lateral and somewhat anterior portions of the brain are injured, in a great many cases the memory is impaired—that is to say, individuals so wounded cannot easily recollect the names of objects with which they have not had a long acquaintance, such as proper names, more especially when they are very compound. We will not attempt to explain a circumstance so singular, but facts exist upon the subject which it is impossible to overlook."

A young soldier had received, at the battle of Eslingen, a ball in the left temple, near the orbit. The faculty of vision was entirely destroyed, the left half of the cranium was more arched and protuberant than the right, and one could perceive both by the eye and touch a portion of the frontal suture, not less than an inch in extent, open by the disunion of the parietal and frontal bones. His senses and almost all the faculties of animal life were extinct. The wound was sounded, the ball, which was discovered, *vers la fosse orbitaire*, was extracted, and the aperture, through which it was withdrawn, exposed to view the pulsations of the brain. After this operation the symptoms began to improve, and as improvement advanced a new language, like that of infants when they first begin to babble, was substituted by the patient for his vernacular tongue. His affirmatives were all expressed by the single word *baba*, his negatives by *lala*, and when he wished for anything he pronounced with some force the words *dada* or *tata*. Being unfortunately seized by fever, he died three months after the extraction of the ball. On inspection the dura mater, which corresponded with the opening between the sutures, was very much thickened and firmly adhered to the edges of the separated bones, the circumvolutions of the subjacent portion of the brain were effaced, and the arachnoid and pia which covered it were much condensed and adhered to each other. An opening existed in that portion of frontal bone, which lies below the left temporal process and behind the external angular apophysis. This hole was from six to seven lines in diameter, its edges were smooth and rounded, and extending from it towards the frontal bump of the same side was discoverable a cicatrized fracture—*une felure cicatrisée*. Other vestiges of ossification were observed, in the indented edges of the separated bones, and in connecting to the os frontis a lamella of bone, which had been originally detached, by a membranous substance which was partly ossified.

A cavalry officer, æt. 26, was wounded in the upper jaw, near the left ala of the nose. The instrument penetrated into the left nasal fossa three inches and a half, traversed the cribriform plate of the ethmoid bone, and entered eight or nine lines at least into the left anterior lobe of the brain, so as nearly to reach the anterior part of the corpus callosum. Very violent hæmorrhage followed the accident, a large quantity of blood was effused into the interior of the craninm, and syncope at length occurred. Every sense was at first abolished, and afterwards only gradually returned. The right eye, right nostril and right half of the tongue, were longer than the left in regaining their activity, and the right side of the body laboured under a general hemiplegic affection, which was very slowly dissipated. The patient's recollection of all substantive nouns, which have any analogy with proper names, was totally extinguished, whilst the memory of images and every thing which was susceptible of description remained perfectly entire. Although for a long time well acquainted with the author, he could not recollect his name, calling him always by that of *M. Chase;* and the names of his nearest relatives were equally forgotton. "The mental aberration, which this officer at first suffered, had ceased, but every thing which referred to himself, or to his history, cast him into a state of melancholy and confusion, whilst conversation, which referred to his family, his parents, or his friends, restored to him the free exercise of his intellectual faculties."

From a case which follows, in which a wound received near the external angle of the left eye, which was followed by a temporary loss of the senses and even some derangement of intellect, it would appear that this apparent defect in recollecting names is less attributable to loss of memory, than to an impracticability in pronouncing them. In this instance the patient could write down the name of any object, or person with which he was acquainted, and if some pieces of money were given him to count, he could tell their number by counting on his fingers.

Tetanic symptoms are frequent consequences of wounds of the head, and although they have been very generally ascribed to some immediate injury done to the brain or nerves, or to the inflammation which such injuries produce, the Baron is disposed to believe that they often arise from laceration of the membranes and fissures of the internal table of the scull.

A young soldier received on his right eyebrow a kick from a horse, which divided the integuments, and fractured the external wall of the frontal sinus. The hæmorrhage, which followed the wound, was great, and all his animal functions were paralyzed. Some hours afterwards, however, he gradually revived, when he began to complain of extremely sharp pains in the wounded part, and convulsive movements of the lips and mouth. In defiance of very active treatment symptoms of phrenitis appeared, and rapidly advanced, general fever and delirium speedily supervened, a state of profound lethargy succeeded to these symptoms, and on the 19th day of the disorder the patient died. The pains, which he experienced in his head during the close of his illness, were so severe that the irritation of even the mildest dressings threw the muscles of the face into convulsions, and while the left arm was palsied the right arm was convulsed as often as convulsions attacked the face. Inspection of the body disclosed to view very intense inflammation and swelling of the mucous lining of the frontal sinus and nasal fossæ, a fissure in the posterior wall of the sinus, so fine as to be almost insensible, intense inflammation of the corresponding part of the dura mater, and some extravasation of blood mixed with serum between this membrane and the right anterior lobe of the brain. The pia mater was equally inflamed and covered with points of suppuration, the substance of the brain, especially that of the right hemisphere, was dense and its vessels were engorged. The mucous surface of the pharynx and larynx was red, the bronchia were filled with reddish-coloured fluid, and the lungs, which were of a brown colour, were filled with blood.

"The rapid progress of these inflammatory and nervous symptoms, (says

Larrey) at first induced me to believe that extreme sensibility of the pituitary membrane had produced them; but succeeding observation has convinced me, that they were mainly attributable to the fissure of the internal table of the frontal bone, and to injury of the fibrous texture of the membranes. I believe, nevertheless, that inflammation of the Sneiderian membrane, by its sympathetic effects upon the entire system of the organic life, contributed much to aggravate that of the meninges, having discovered in the air-passages all the traces of the same inflammation. It is likewise not without reason that the ancients regarded wounds of the frontal sinuses as very dangerous, especially when air entered the nasal fossæ, and it is on this account that they recommend the exclusion of such wounds, and every precaution which may prevent the exposure of the fine and sensible lining of the olfactory labyrinths to the air."

A cuirassier was struck by a horse so violently on the right eyebrow, that the external wall of the frontal sinus was broken to pieces, active hæmorrhage both from his nose and ears occurred, and he lay as dead upon the ground. When visited by Larrey he was insensible, his head was turned to the left shoulder, the body was of an icy coldness, and tetanic symptoms of the right side were manifest. The head was shaved, embrocations of warm camphorated vinegar were applied to the body, the loose portions of bone were extracted, and some clots of blood, which had been enclosed within the sinus, were removed. During this operation the patient bled freely by the nose and wound, and scarcely was the first dressing terminated before his senses and intellect were restored. Ice to the head, stimulating injections, diluents and other antiphlogistic remedies were employed, and as the pulse became strong and the temperature of the surface rose, a large abstraction of blood from the arm was practised. But, although all these means were assiduously employed, although the depletion was more than once repeated, pains in the occiput and in the region of the wound came on. These were somewhat appeased by making a deep incision into the integuments on the right side of the occiput, which were œdematous; but the right eye was lost, and about the 20th day nervous excitement appeared, some tetanic convulsions of the two extremities of the wounded side came on, and deep-seated pains were complained of along the entire right side of the head, which became so severe that this part could not be touched, even in the mildest way, without producing horripilation, convulsions and intense agony. Blood was successively taken from the arm, neck and foot, many cupping-glasses were applied to the nape, shoulders and spine, and ice to the head and stimulating baths to the lower extremities were continued. After some transient relief, the tetanic symptoms augmented, the right testicle swelled and gave excruciating pain, and, what is more remarkable, the hair and whisker belonging to the right side of the head bristled, and imparted a sensation of the most lively pain on the slightest touch. He was brought before the *Societé de Medicine de la Faculté* of Paris, and, although his hair was cut by the very best edged scissars, horripilation, convulsions and a general painful tremor of the parts convulsed, extending even to the toes and fingers, followed the experiment. The author ascribed all these symptoms to a fissure of the right side of the occipital bone, to laceration of the subjacent dura mater, and to effusion either underneath this membrane, or the right posterior lobe of the brain. But as the patient left the hospital seven months after his accident, the sequel of his case is not given.

"We have hitherto devoted ourselves to a consideration of the differences, which exist between the lesions of different portions of brain, and the particular phenomena which characterize each of these lesions, with the view of establishing an accurate prognosis, and of indicating in a more precise manner, than has hitherto been done, the therapeutic means which are best adapted to their treatment. We shall now proceed to treat of such injuries of the head, as require the operation by trepan; 2dly, of such, on the contrary, as cannot be improved, but may be injured by this operation, in opposition to the assertions of many authors; 3dly, of the treatment necessary in cases of hernia of the brain; and lastly, of the causes of abscess of the liver after wounds of the head."

In elucidation of the first point eight very interesting cases are detailed. In the 1st, a soldier received a ball in the middle of his forehead, which pierced the bone, and, traversing along the left side of the longitudinal sinus, penetrated even to the occipital suture, where it stopped. By means of the crown of a large sized trepan a counter opening was made in the occipital bone, the ball was extracted, and a large quantity of clotted blood, mixed with purulent matter, escaped by the opening. The cure went on uninterruptedly, and the patient recovered. In the 2d case the ball penetrated the scull at the left parietal bump, grazed the internal surface of the parietal bone, and lodged about half an inch from the occipital suture. The trepan was applied over this latter part, the ball, which was partially encrusted with bone, was removed, fifteen days passed without being marked by any disagreeable symptoms, and had not the patient been unexpectedly seized with fever, there was no reason for anticipating any other than a favourable result. "These two cases," says the Baron, "prove, in opposition to the generally admitted opinion, that to search after foreign bodies within the cranium is not always a useless nor even a dangerous practice, when it is done prudently and with adroitness." In the 5th case a cuirassier was struck on the head by his horse, which had been newly shod. The stroke was so violent as to fell him to the earth, deprive him of sense and envelop him in blood. He was, however, immediately removed to the hospital of Gros-Caillou under Larrey's care, who had his head shaved and examined the wound, which was found to occupy the superior part of the right temple. The soft parts, covering this region, were divided for the space of two inches and a quarter, the temporal portion of the frontal bone was fractured, and some splinters of it were depressed. As, however, there were no symptoms of compression present at this period, the author contented himself with cutting freely down upon the injured bone, so as to expose the fracture—with detaching, by means of the rasp, that part of the pericranium which covered the fractured bone—with tying a few branches of the temporal and frontal arteries which bled, and with dressing the wound with adhesive plaister, charpee, a square compress and Galen's bandage. The patient was bled from the arm, sinapisms were applied to the feet, ice to the head, and cold diluent drinks were freely ordered. During the night he was bled from both the jugular vein and arm, and on the next day he was comatose and laboured under hemiplegia of the left side. The pulse was small, and so slow as to number 45 strokes only in the minute; the bowels were confined, the bladder was incontinent, and the feet were cold. The dressings being removed it was attempted to raise the fractured portions of bone, but in vain, and the trepan was determined on as the only remaining hope. The fractured bone and about a spoonful of black blood, which had been effused between the dura mater and cranium, were removed by the operation, and scarcely had it been finished when the patient was able to reply to any questions addressed to him, his pulse rose to 55, and in a few hours afterwards his hemiplegic symptoms disappeared. The hole made in the scull by the crown of the trepan, was filled with fine washed sponge—a practice which the Baron always follows;—light dressings were placed over it, and the remedies in former use were continued. On the ninth day all danger was gone, and he was afterwards presented to the Academy of Surgery as a man indebted for his life to the operation by trepan. The 7th case is very interesting, as strongly confirmatory of Larrey's doctrine, that "the trepan should be immediately employed in all fractures of the bones of the cranium, attended with depression of the fractured portion, injury, or depression of the dura mater and brain." It is, however, so long, and so much important matter still remains unnoticed, that we can thus merely draw the attention to it while passing.

Most authors have been unfavourable to the practice of applying the trepan over the frontal sinuses, as well as along the track of the meningeal arteries, on account of the indeterminate depth of these cavities, and the aerial fistulæ which they conceived afterwards to occur and fancied to be fatal. The author has, however, deviated from the old and ordinary custom, and has adopted this practice in two cases. The operation in each case was followed with success.

In defence of his second proposition—that the trepan is not only useless, but injurious in some cases where it is very generally recommended—he observes that—

"Already, while considering the first proposition, we have specified, as one among the most serious exceptions to the operation by trepan, cases in which strange bodies, although introduced into the cranium, lose themselves in the substance of the brain. Of a similar nature are all those cases, where effused fluids either lie at a great distance from the cranial vault, or whose seat is entirely unknown. The trepan is likewise inadmissible in all wounds of the head, complicated with fracture of the skull, it matters not how extended the fracture, or how multiplied its rays, if the fractured portion of the bone be not depressed, and if no foreign body nor symptoms of compression appear. The cerebral disturbance is generally less in extensive wounds, attended with loss of integument and fracture of the bone, because the effects of the percussion are more confined to the external parts, especially when the stroke is directed in the diagonal of the cranial vault. In consequence of this circumstance the internal organs are saved, absorption of the effused fluids proceeds more rapidly, the fractured pieces of bone gradually re-unite, and the patient gets well by the unaided exertions of his own constitution. In such a case, therefore, the trepan, without doing any good, can only retard the cure."

Without attempting to give a definite solution to the question, whence arises *hernia of the brain*, Larrey observes that, when a portion of the brain is exposed by the trepan or an accidental fracture and removal of bone, it is deprived of that support and pressure which are natural to it, and in consequence of its spontaneous expansion, from its innumerable blood-vessels, this portion of brain tumefies, gradually rises through this artificial opening, and ultimately appearing at or above the surface of the bone occasions hernia. This extra-cranial portion of brain then begins to increase, and soon exhibits all the properties of vital erectility, refuses to be checked by compression, or to be controlled by the knife. If compressed, excruciating pain is generally produced in the region of the wound, and if long-continued, vomiting, convulsions, and syncope supervene. If amputated, the cut surface soon furnishes a fresh exuberance, which is merely encouraged in its reproductive process by repeating the amputation. In treating all such cases of disease, it is indispensable to consider that extensive hernia of the brain is one of the most formidable accidents which can accompany wounds of the head, and that those rarely recover who are attacked by it. The existence of hernia implies an extremely irritable state of the pia mater and cerebral vessels, as well as of a deep-seated inflammation in the substance of the brain, which it is difficult, if not impossible to remedy. With the exception of a single case, in which the extra-cranial portion was small, the Baron observed that all whom he ever saw with this affection perished, and the treatment which he recommends is, we fear, little calculated to encourage his readers to form a more favourable prognosis. It consists in—

"Applying to the protruded portion of brain a piece of fine linen, moistened with oil of camomile slightly camphorated, and in removing every cause of excitement, whether external, or within the scull. With this view it is necessary to extract with proper care all foreign bodies, to preserve the injured parts from the air, to prescribe an antiphlogistic regimen, and to employ such dressings as are mild, and cannot by their weight exercise any injurious pressure. When the encephalocele is susceptible of reduction, Nature then aided will herself gradually effect it, and the protruded portion of brain will gradually re-enter the interior of the cranium, like the epiploon when it has escaped through a wound in the abdomen."

Why abscesses of the liver should so frequently succeed to injuries of the head, is a question which has been often agitated, and perhaps never yet satisfactorily answered. According to some they are regarded as accidental complications, having no etiological connexion with the wound of the head, but the majority have been disposed to ascribe them to the concussion and violence sustained by the liver at the moment the injury is inflicted upon the head. This latter doctrine is supposed to be defended by many arguments, and perhaps by none more strongly than by the fact, that such abscesses seldom or never occur when the wounds have been produced by a cause, which confined its operation exclusively to the head, whereas they usually succeed to injuries, which have been inflicted with so much violence as to agitate and disturb the entire system. Larrey is, however, equally opposed to both these views, and is disposed to ascribe to sympathetic irritation of the liver those purulent formations, which occasionally complicate injuries of the head. He has often had occasion to observe, that the pulmonic and more especially the biliary systems were disturbed in their functions, and seriously affected by inflammation of the fibrous membranes of the head, or extremities, particularly of those which correspond with the organs situated on their side. The irritation, established in some portion of these membranes, rapidly propagates itself by sympathy, and the viscera, which are supplied by nerves from the great intercostal seems to be most obnoxious to the effects of this sympathetic irritation. The vital properties become disturbed, inflammation more or less rapidly appears, abscess at length forms, and when once formed co-operates with the original injury in effecting the patient's downfall.

But it is not exclusively after injuries of the head that these disorders in the hepatic organ arise. According to the present writer they are equally frequent after wounds of the ginglymoid joints of the extremities; and neither is the liver the only organ in which abscesses form after wounds of the head, for every other viscus, whether in the chest or abdomen, may also receive the communicated irritation, and present analogous derangements. The conclusions, then, to which the Baron arrives are—

"1st. That these abscesses rarely recognize, for their essential cause, any direct violence inflicted on the liver by the fall of the individual, or from the action of any body, which may have struck the right hypochondre. 2dly. That the cause of these abscesses after injuries of the head should be referred to the sympathetic irritation, which this organ receives from the inflammation established in the fibrous membranes of the cranium, or of the bones of the upper and inferior extremities, especially of those of the same side, and to a metastasis of the *ichorous miasmata,* or acrid fluids to this organ: and 3dly. That these communications of morbid principle between the liver and part originally injured are more easily effected, when they are not obliged to traverse the median line of the body."

Passing over the author's section on *Apoplexy,* as it contains nothing of importance, *Injuries and Diseases of the Cerebellum* are the next subjects which are treated, and perhaps there is no portion of the volume which merits more attention. Experience has taught the Baron that active inflammation of the cerebellum, whether arising spontaneously or by accident, is constantly followed by an exaltation of the animal and organic sensibilities, without producing the slightest disturbance in the intellectual operations. When one only of its lobes is inflamed, this increase of sensibility is manifested in the same side with the injury, lively pains are felt in the occiput, horripilations, contractions of the muscles of the face and of the two extremities belonging to the wounded side, with a sort of painful formication in the toes and fingers are complained of. Vision is often disturbed, the horizontal position is preferred, forced extension of the spine produces convulsions and sometimes alarming syncope, and these tokens of deranged sensibility are more frequently manifested in the posterior than anterior surface of the body. If the inflammation terminate in suppuration, it is generally established immediately beneath the pia mater. The arachnoid loses its transparency and becomes thickened, but is without any red vessels; and by degrees the purulent matter extends into the proper substance of the cerebellum, and ultimately as far as the arbor vitæ. Feverish excitement, with transient apyrexial intervals, a dull and fixed pain in the occiput, a sense of weight in the head, loss of hearing in the affected side, absence of speech or difficult pronunciation, are among the most frequent symptoms, by which this morbid condition is recognized. If the cause, which alters the substance of the cerebellum, does not compress the nerves of the medulla oblongata, there is no paralysis, and the genital organs alone receive the effects of this morbid state; and should even the patient recover, which is a very rare occurrence, he is ever after annoyed with a feeling of excessive tenderness in the nape and occiput, asthenia of the genital organs, with atrophy of the testicle which corresponds to the diseased lobe of the cerebellum, or of both testicles, if the whole of this portion of the encephalon be affected.

Singular as these effects of diseased cerebellum upon the organs and functions of generation must appear, it is still more remarkable to observe the influence which diseased testicle exerts upon the growth and development of the cerebellum. If the Baron's statements and cases are to be depended on, and they are not quite solitary nor unsupported, it would appear that these organs—the cerebellum and testicles—act and re-act on each other, that disorder or disease in one disorders and deranges the other, and that the effects thus mutually exerted are in a direct ratio to the extent and inveteracy of their disease.

"The genital organs seem to have a marked influence upon the cerebellum, for when they are removed by disease, or any other means, the occipital region of the cranium and cerebellum gradually experiences such a sensible reduction, that the occipital bumps, which had been more or less protuberant before, disappear, and the whole occipital region of the head is diminished in proportion. We have verified this change of dimension in a great number of soldiers, who had been operated upon for sarcocele, and when one testicle only was removed there was only a reduction of that portion of the cerebellum and occipital bump, which belongs to the same side, with the extirpated testicle."

A soldier, who had been wounded in the occipital region by a splinter of wood, was attacked with all the symptoms of inflamed cerebellum, and, in despite of every thing which was done, they were only dissipated by the appearance of an abscess in the nape, which opened spontaneously. In about three months after the accident he rejoined his regiment, and many years elapsed before he again came under Larrey's notice. He was then so extremely altered in appearance, that the author mistook him for a young conscript, who had been exhausted by some asthenic disease. He was 32 years of age, of middle size, but thin and pale, his eyes were depressed, his lips blanched, his hair, more especially that which covered his occiput, was thin and bristled, and a feeling of pain and coldness was always experienced in the back part of his head. He was beardless, his voice was feminine, and as some of the assistants were not without suspicion of his sex, a more minute examination was considered necessary.

"To our great surprise, (says Larrey,) we found his genital organs reduced to the size of those of an infant some months old. His penis was not more than five or six lines long, and two or three lines thick, it never exhibited any degree of erection, and his testicles were so wasted as scarcely to equal in size a small bean."

A Swiss soldier of the guards, named Granfort, fifty years of age, was received into hospital for an erysipelatous affection of the left side of the face, attended with habitual pain and weight of head, deafness of the left ear, and a great difficulty of speech. The pulse was febrile and the strength was much reduced. These symptoms were occasioned by a fall, which this man had sustained a few days before the appearance of the erysipelas. Emollients were externally applied; diluents and anodynes were internally administered. After some days fluctuation was felt, and a deep incision discovered a large abscess in the neighbourhood of the left mastoid process, which was denuded and carious in one point, where a communication had been formed between the internal ear, which was the seat of the abscess, and the external surface upon which it pointed. Five or six weeks afterwards the walls of this abscess were clean and began to heal; still, however, pains in the occiput, a sense of weight in the head, and considerable difficulty in keeping the head from falling towards the affected side, were complained of. He seldom spoke, and when he did he articulated badly. The integuments covering the occiput were very sensible, the arm and hand of the left side were threatened with palsy, but his mind was completely undisturbed. After two months convalescence this soldier fell into a state of lethargy, and died twenty-four hours afterwards. On inspection the dura mater appeared of a deep brown, the arachnoid was opaque and in some parts of a dull white, these membranes and the cerebral mass were filled with turgid blood-vessels, the consistence of the brain was firmer than natural, the lateral ventricles contained some colourless fluid, three spoonsful of pus were found in the cerebellum, the right lobe was diminished, and the medullary substance forming the arbor vitæ was dense and of a grey color. The purulent matter was effused underneath the pons varoli and into the lambdoidal fossa, where the carious opening lay which communicated with the internal ear, and around which the membranes had contracted adhesions. "The scrotum and penis were so reduced from their primitive volume, that one might consider them as being in the second stage of atrophy."

John Baptist Dandé, aged 26, of a scrofulous habit, who had been formerly under treatment for diseased spine, was attacked with pains in the left testicle, which swelled and obliged him to apply for relief. It was at first regarded as a consequence of suppressed gonorrhœa, although the soldier denied that he ever had any syphilitic symptoms, and he was treated in accordance with this view. The tumefaction of the testicle increased however, and extirpation of this organ was at length determined on. After two months the cure was supposed to be complete, and Dandé rejoined his regiment; but in about six months afterwards the other testicle became attacked with symptoms of a similar character to those which had been experienced formerly, and every effort to resolve the swelling and to save the organ being made in vain, the appearance of symptomatic fever and other malignant tokens of inveterate disease suggested the necessity of a second operation. No disagreeable symptoms followed—

"But, one remarkable thing is, that the nape now appears sensibly depressed, that the occipital bump, corresponding with the testicle first amputated, is much smaller than the right. The body is likewise emaciated, the beard and mustaches are nearly fallen off, and, in fine, it is obvious that the total loss of the genital organs has had a very marked effect upon the cerebellum, since the occipital region offers a profound and anormal depression—the consequence of atrophy, which also affected all the bones of the scull, the skin covering the face, and the beard."

These are most interesting cases, and the physiological as well as practical inferences, which arise from them, are of the first importance. We regret that space does not permit us to extract three or four others, which are of the same description; but we think that the intimate relationship which exists between the cerebellum and generative organs, both in health and disease, is incontestably established. Before leaving this subject we may observe that, from some experiments which have been made upon inferior animals, there is reason to believe that the decrease, which the occipital region suffers by castration, is sometimes attended by a corresponding increase in the frontal region. How far this anterior augmentation of cerebral capacity is connected with and influences the intellectual functions, the nature of the animals, which have formed the subjects of experiment, prevents all but the phrenologist from hazarding a conjecture.

Larrey's theory of *nostalgia* is novel. According to him the mental faculties are always the first affected in this disease and derangement of the senses follows. The head gets warm, the pulse becomes strong, the eyes red, the speech precipitate and inaccurate, dull pains are felt in different parts of the body, and the bowels are constipated. To this feverish state succeed symptoms of compression and collapse; and, finally, asthenia declares itself with sad prostration of every mental, moral and physical faculty. On inspecting the bodies of those, who have died of this disease, the Baron has found the surface of the anterior lobes of the brain inflamed, with points of suppuration of variable extent. The arachnoid and pia mater were in a similar state, the cerebral substance was hardened, and its vessels were filled with black blood. The lungs were engorged, the cavities of the heart were occupied by black coagulated blood, the stomach and intestines were distended with gas, their mucous lining was injected, but not inflamed. Ossification, he thinks, of the sutures of the cranium and of the arteries of the encephalon predisposes to this disease, and its proximate cause he is inclined to ascribe to chronic cephalitis, gradually developed under the influence of a strong moral affection. The celebrated Fourcroy died of this distemper, and Lord Byron is supposed by Larrey to have sunk under a form of fever, which seemed to hold its seat in the brain, and to have a strong resemblance to nostalgia. The necropsy of Byron is similar, no doubt, to that of the nostalgic dissections which he gives as marking this affection. The thickened and suturless scull, the firm adherency of the dura mater to its internal surface, the injected condition of the cerebral vessels, and the large quantity of serum which occupied the cavities of the brain—are all indicative of cerebral action; but it is well known that this illustrious man always expressed himself happier when at a distance from his country, than when at home.

For the treatment of this wretched and obstinate affection the author recommends moderate depletion, constant occupation both of mind and body, variety of business and amusement, change of scenery, enlivening music and agreeable society. Vigarous, a Professor at Montpellier, was accustomed to cure all our hippish countrymen, who consulted him, by enjoining them to take active exercise, and by giving them medicated *placebos* at an extravagant price. Our German Spas, our Cheltenhams, and our Buxtons owe much of their efficacy on invalid constitutions to the pure air, active exercise, agreeable society and total relief from anxious occupations, which such places furnish to the invalid; and even a trip to Gravesend, or a walk to Richmond is often more efficacious than the prescriptions and purgatives of half a year.

Some curious physiological observations are made on the nerves of animal or exterior life, and on the resemblances which are observable between them and the *electrical telegraph* of Soemmering. This telegraph consists of about 35 fine metallic wires, which are connected and communicate with what Soemmering calls the *interrogator* and *respondent* of the instrument. The *interrogator* is composed of a series of horizontal metallic cross bars, separated by equal distances, and distinguished by being marked at one end with the different letters of the alphabet. Each of these bars has at one of its extremities one aperture, large enough to admit the points of two electrical conductors, and another much smaller for the passage of a metallic wire. These wires, at first separated at their points of insertion, and isolated in their whole extent by threads of silk, are afterwards brought into juxta-position and variously intertwined, so as to form one common cord; and, after running along for some space in this form, they again separate, diverge and anastomose or articulate with points of gold, which are vertically placed in a square glass vessel, filled with pure water, which is called the *respondent.* The same letters, which mark the cross-bars of the interrogator, are also placed over these points of gold in the respondent. When electric or galvanic fluid is transmitted by means of the two conductors, coming from the two poles along these metallic wires, it is prevented by the intermediate silk threads from passing through one wire to another, and goes directly forward to render itself at the corresponding gold points in the respondent. The electricity, which issues from the negative pole, disengages hydrogen from the water in the jar, and that proceeding from the positive pole produces a similar disengagement of oxygen. These gases, rising in bubbles to the surface of the water, are received into gazometers, so that it is easy to determine the nature and the quantity of the gases generated in a given period of time. Now, says Larrey, the nerves of animal life are disposed in the same manner, and present in their functions analogous phenomena. For example, the seventh pair consist of two distinct nervous cords. The auditory portion arises by two or more small fibrillæ, from one part of the brain; the facial portion arises from another. The former, more elevated towards the positive pole of the medulla oblongata, is destined to form the organ of hearing; the latter, being nearer the negative pole, is destined to supply the muscles of the face with motive power and the skin and glands with sensibility. A similar disposition holds with the lingual nerve, of which one part serves for taste and another for speech, each having a different origin. The branches of the median nerve, which supply the muscles of the arm, fore-arm and hand, do not arise from the same part of the spinal marrow with those which it sends off to endow the skin with sensibility. The neurilema acts the same part to the nervous fibrillæ, which the silk threads do to the wires of the telegraph; and the common membranous sheath, which surrounds the nervous trunk, represents the silk ribbon which envelops the metallic cord of this instrument: so that the electrical telegraph of Soemmering, according to Larrey, may be styled the *simulacrum nervorum.*

"All this proves that the intellectual and sensitive functions are executed by distinct agents, which give different results, although they communicate and sympathize. Thus, we shall suppose that the nerves, which arise from the negative pole of such or such a pile, give motive power to the muscles or conductors which are of a stronger and more active principle. To take for an example the pile of the medulla oblongata, which has for its base the centre of the medullary substance of the brain, I believe that all the nerves of sensation arise from the superior part of the positive pole, which in Soemmering's telegraph disengages oxygen—such are the olfactory, the auditory, the optic nerves. Whilst those, which supply functions requiring greater intensity of power, such as those of the muscular tissue, take their origin from a more inferior part of the medulla, or towards the negative pole, which in the telegraph produces hydrogen."

How much truth is mixed up with this theory of the differences between nerves of sense and motion, it would require more time and consideration to ascertain, than the present place can furnish; but we cannot but express regret, that not the faintest allusion is made throughout all this discussion to the ingenious discoverer of the differences themselves. Larrey has, surely, no reason to harbour jealousy against either nations or individuals. He has been amply rewarded by his country for his zeal and success in advancing surgery; and he will not the less enjoy the harvest of his labours, by respecting the claims of Bell, or any other celebrated *exotic*, whose improvements are not the fewer, because their remunerations have been more economically awarded.

After a few observations on some lesions of the ear, and a rather operose description of the conjunctiva of the eye, the author divides the morbid alterations, which the globe of the eye sustains, into such as are produced by concussion, and actual solutions of continuity.

A brigade officer, during the battle of Aboukir, was struck by a ball on the external side of the right orbit, which so disturbed the right eye, without injuring the external skin, that it was wholly deprived of its visual faculty. A severe pain was felt at the bottom of the orbit, followed by weight of head, effusion of blood into the chambers of the eye, and engorgement of the conjunctiva. After topical bleeding and other local measures, Larrey gave exit to the extravasated blood within the eye by an operation; the patient immediately saw the light without being able to distinguish objects, every symptom gradually disappeared, and the cornea healed without opacity, or any sensible deformity.

The following case is very singular. Pêcheur, a serjeant in the Royal Guards, received so violent an injury on the left eye by a fall, that the globe of the eye was burst, and the crystalline lens, together with the aqueous and vitreous humours, were forcibly expelled. The palpebræ were ecchymosed, the conjunctiva was red and bloated, and the lower part of the cornea was separated from its attachment to the sclerotic by an irregular cut, through which a portion of the iris protruded. The parts were washed, the iris was carefully returned, the divided edges of the cornea were approximated, the eyelids were drawn close and retained so, and a quantity of blood was drawn from the temporal artery. Ice to the head, sinapisms to the feet, constant darkness, and a rigorous diet were enjoined. During the night a second bleeding was practised, and the next day cupping-glasses were applied to the nape and between the shoulders.

The Baron had very naturally, it will be admitted, considered this eye as lost, or at least for ever deprived of the faculty of sight; but, *a notre très-grande et agreable surprise*, the globe gradually refilled, on the 22d day the cornea began to assume a healing aspect, and in about six weeks it was perfectly cicatrized. The natural form and almost ordinary volume of the organ were restored, and by the aid of a very convex glass this soldier came to see objects distinctly, and continues to discharge all the duties of his office. "This very curious case evidently proves, that the vitreous humour, although in greater part lost, can be regenerated," and that the crystalline lens may be dispensed with!

It has been very generally believed that the *iris* derives its contractile power from the influence of the optic nerve, or retina, and hence has it been recommended not to operate for cataract, especially when extraction is necessary, if the iris appear motionless. The author, however, opposes this doctrine by arguments numerous, if not convincing, and contends that the properties of this membrane depend exclusively on its own texture, and on the ciliary nerves which are sent to it from the lenticular ganglion of the great sympathetic. Cases of amaurosis, he argues, are met with in which the iris retains its contractility—wounds inflicted on the nerves, by which its mobility is supplied, have palsied this membrane, while the faculty of sight remained unaffected, and belladonna suspends its contractility without disturbing any other function of the eye;—these and other arguments, which are illustrated by cases, but to which we can only refer, are urged by Larrey to prove that the iris is independent in all its functions of the optic nerve.

*Ophthalmia*, and especially the endemic ophthalmia of Egypt, is the next disease treated of. With the exception of the gonorrhœal form of this disorder, he strenuously contends for its non-contagious nature, and considers it important both to the interests of science and to public tranquillity, to dissipate any impression which may exist of a contrary character; and with some ingenious remarks upon the causes, varieties and treatment of *epilepsy*, he concludes one of the most interesting and valuable volumes, which for a long time we have had the pleasure of perusing. As opportunity offers, we shall not neglect to draw the attention of our readers to the remaining portions of the *Clinique Chirurgicale;* and should we find them executed with the same talent and success, by which the present is characterized, the name of Larrey, justly honoured and highly exalted as it already stands, will go down to posterity with purer claims on their esteem and gratitude, than any which can be presented by that of the mighty and almost masterless conqueror under whom he served.

